# Sexual health—a topic for cancer patients receiving oncological treatment with palliative intent

**DOI:** 10.1186/s12904-024-01513-4

**Published:** 2024-07-29

**Authors:** Claudia Schmalz, Anne S. Oberguggenberger, Eva Nagele, Brigitte Bliem, Anne Lanceley, Andy Nordin, Karin Kuljanic, Pernille T. Jensen, Vesna Bjelic-Radisic, Alexander Fabian, Juan I. Arraras, Chie Wei-Chu, Carien L. Creutzberg, Razvan Galalae, Hilde Toelen, Kristin Zimmermann, Anna Costantini, Thierry Almont, Samantha Serpentini, Ligita Paskeviciute Frøding, Ingvild Vistad, Krzysztof A. Tomaszewski, Elisabeth Inwald, Elfriede Greimel, the EORTC Quality of Life Group

**Affiliations:** 1 Department for Radiation Oncology, University Clinic Schleswig Holstein, Campus Kiel, Arnold-Heller Str. 3, Kiel, 24105 Germany; 2 Medical University of Graz, Graz, Austria; 3 University College London, London, UK; 4 Medical University of Innsbruck, Innsbruck, Austria; 5 East Kent Gynaecological Oncology Centre, Elizabeth the Queen Mother Hospital, Margate Kent, Queen CT94AN UK; 6 Clinical University Hospital Centre Rijeka, Rijeka, Croatia; 7 Department of Gynecology, Aarhus University Hospital, Aarhus, Denmark; 8 Helios University Clinic, University Witten/Herdecke, Wuppertal, Germany; 9 Complejo Hospitalario de Navarra, Pamplona, Spain; 10 Institute of Epidemiology and Preventive Medicine, National Taiwan University, Taipei, 100 Taiwan; 11 Department of Radiation Oncology, Leiden University Medical Center, Leiden, Netherlands; 12 Department for Radiation Oncology, Klinikum Bremerhaven, Bremerhaven, Germany; 13 University Hospitals Leuven, UZ Campus Gasthuisberg, Louvain, Belgium; 14 Bundeswehrzentralkrankenhaus Koblenz, Klinik Für Urologie, Koblenz, Germany; 15 Psychoncology Unit, Sant’Andrea Universitary Hospital, Rome, Italy; 16 Institut Universitaire du Cancer, Toulouse, France; 17 Veneto Institute of Oncology IOV – IRCCS, Padua, Italy; 18 Department of Gynecology, Copenhagen University Hospital, Copenhagen, Denmark; 19 Sørlandet Hospital, Kristiansand, Norway; 20 Faculty of Medicine and Health Sciences, Andrzej Frycz Modrzewski Krako´W University, Krako´W, Poland; 21 University Medical Center Regensburg, Regensburg, Germany

**Keywords:** Patient reported outcome measure, Sexuality, Patient satisfaction, Neoplasms, Palliative care

## Abstract

**Objectives:**

Sexuality is an important dimension of health-related quality of life (HRQOL) in cancer patients. Studies evidence that most patients report impairments of their sexual health related to their disease or its treatment. The Quality of Life Group of the European Organization for the Research and Treatment of Cancer (EORTC) developed a patient reported outcome measure assessing multidimensional aspects of sexual health. The validation study for this instrument revealed heterogenous results for patients in palliative oncological treatment. The aim of this secondary analyses is to examine differences in patient related sexual health outcomes between palliative patients with good performance status (GPS) and those with poor performance status (PPS).

**Methods:**

In this observational cohort study, self-reported sexual health issue scores were compared between the two groups of patients in palliative oncological treatment with GPS vs PPS status.

**Results:**

Patients with GPS experienced significantly more sexual satisfaction than patients with PPS (*p* = 0.015). They reported significantly more treatment effects on their sexual activity (*p* = 0.005) and suffer more from decreased libido (*p* = 0.008). Patients with PPS reported significantly more fatigue (*p* = 0.03) and regarded preservation of sexual activity of higher importance than did patients with GPS (*p* = 0.049).

**Conclusions:**

Our study demonstrates the importance of sexuality for patients in palliative oncological treatment, especially for those with limited performance status. Considering the patients´ perspective, sexual health reaches beyond physical functioning. Patients in a palliative phase of disease report high levels of psychosexual problems while sexual performance deteriorates. Sexuality is an important aspect of HRQOL for these patients, needs to be addressed by health care providers and sensitively integrated into palliative care plans.

## Objectives

Sexuality is an important dimension of health-related quality of life (HRQOL) in cancer patients. Consequences of cancer disease and treatment on sexual health can be observed not only during the treatment phase but persisting into survivorship. Recently, Falk and Dizon [1] demonstrated that between 35 to 94% of female cancer survivors and 40 to 49% of male cancer survivors experience impairments in body image and sexual functioning caused by the illness or its treatment. There is limited evidence regarding the impact of cancer on sexual health in palliative treatment settings; individual studies have indicated that, for example, physical limitations, disfigurement or the need for aids can play an important role in this context alongside psychosocial factors [2, 3, 4]. In line with Greimel et al., we used a broad definition of sexual health as a multidimensional construct, including physical, socio-behavioural and psychosocial dimensions of sexuality [6].

Despite the prevalence of up to 94%, sexual problems are often not identified during routine clinic appointments [5]. To address this problem, the Quality of Life Group (QLG) of the European Organization for the Research and Treatment of Cancer (EORTC) developed a patient reported outcome measure to assess the multidimensional aspects of sexual health, including physical and psychosocial dimensions of sexuality [EORTC QLQ-SH22, [6]]. The psychometric validation of the questionnaire demonstrated that patients with cancer undergoing treatment with curative intention have significantly higher scores on sexual activity and sexual satisfaction scales than patients undergoing treatment with palliative intent. However, results reported in the palliative treatment group were notably heterogenous. Furthermore, the study revealed a clear statistical correlation between performance status and sexual health: The study reported significant differences between patients with a higher Eastern Cooperative Oncology Group (ECOG) performance status and those with poor ECOG performance status concerning sexual satisfaction, treatment effects on sexual activity, libido and fatigue [6]. In line with clinical expectations, patients with poor performance status reported lower sexual satisfaction and significantly lower libido, and experienced more fatigue and more treatment effects on their sexuality.

There is some evidence that many patients undergoing oncological therapy with palliative intent have a moderate or good performance status rather than a low status [7]. Recent studies demonstrate that differences in ECOG performance status may have a predictive impact on survival in different groups of palliative patients [8, 9, 10]. The aforementioned studies showed that an ECOG status of at least 2 for patients in outpatient palliative care and for patients undergoing palliative irradiation is a differentiating criterion with regard to overall survival. Individuals living longer with advanced cancer are challenged to engage in life and intimate relations while living with symptomatic disease and closeness to death. This circumstance may dramatically alter intimate relations but does not necessarily diminish the importance of sexual health and intimacy for the person. Studies exploring sexual health in palliative patients are generally lacking.

### Aim

This observational cohort study aims to evaluate differences in patient reported sexual health outcomes between patients in oncological treatment with palliative intent with GPS vs PPS. We hypothesized that there may be differences in patient related sexual health outcomes between palliative patients with GPS (= ECOG 0–1) and patients with PPS (= ECOG 2–3).

## Methods

**Fig. 1:**
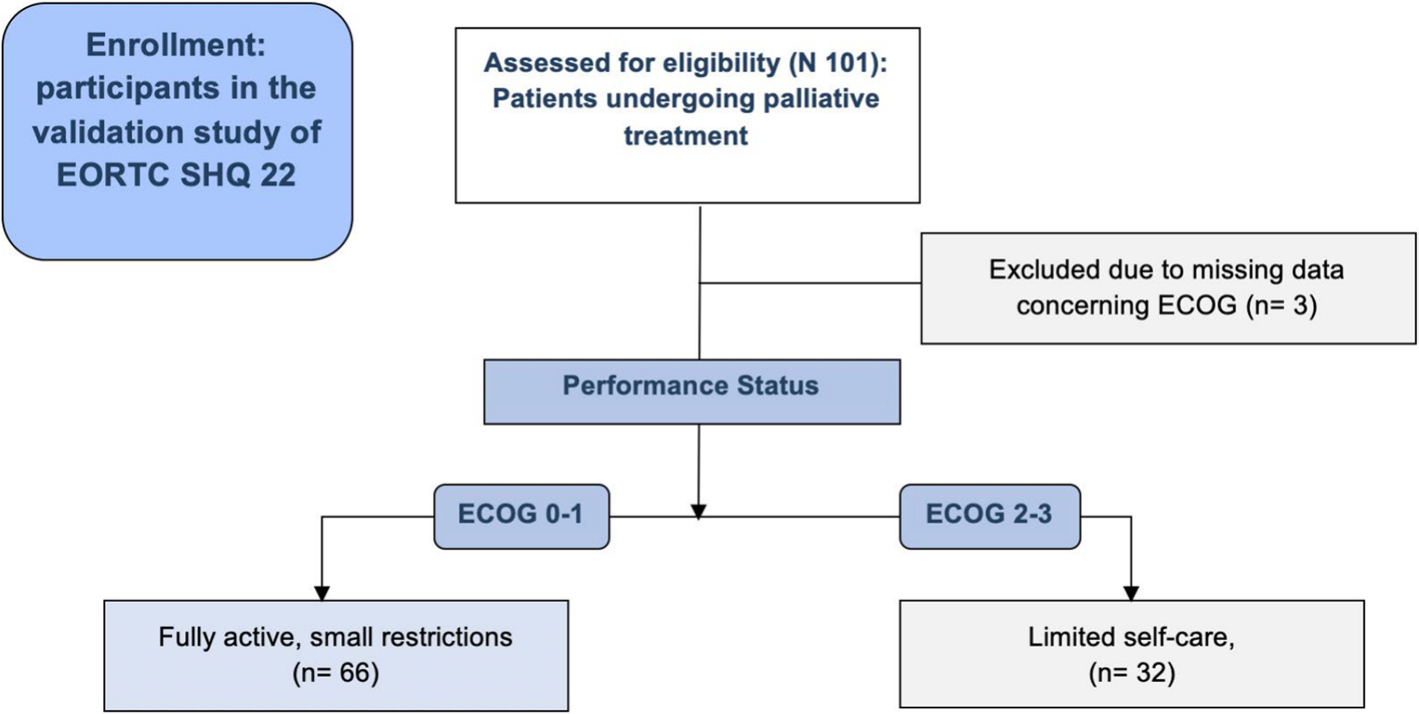
Flow diagram: enrollment of patients

The study is a secondary analysis of the international validation study of the EORTC QLQ-SH22 [6] using a subsample of patients undergoing oncological treatment with palliative intent (see Fig. 1). As in the validation study, palliative treatment intent was defined as second- or third-line oncological treatment with or without surgery. In the validation study the newly developed 22-item questionnaire for assessing sexual health issues in cancer patients and survivors was psychometrically tested. Patients eligibility criteria were histologically diagnoses of cancer, any tumour site, no cognitive impairment and 18 years of age and above. A sample of 444 cancer patients from 18 collaborating institutions in 13 countries across Europe and Taiwan participated and completed the EORTC QLQ-SH22 in different status of the illness (curative, palliative and survivorship). The questionnaire consists of two multi-item scales assessing sexual satisfaction and sexual pain and 11 single items. All items are scored on a four-point Likert scale from 1 to 4 (not at all, a little, quite a bit and very much). Higher QLQ-SH22 scores in the multi-item scale for sexual satisfaction/ communication/ confidence indicates a higher level of sexual satisfaction/ communication/ confidence. A high score for all other multi-item scales and single items represent a higher level of symptom burden. The scores of QLQ-SH22 were linearly transformed to a 0—100 scale according to the scoring manual of the EORTC QLG [11]. The EORTC-QLQ-SH22 includes five partner-related items and four gender- specific items. Scale structure, clinical validity, and statistical analysis of the EORTC-QLQ-SH22 has been previously published [6].

Patients in oncological treatment who participated in the validation study treated with palliative intent were eligible for the present analysis. The study sample was divided into two subgroups: GPS group including palliative patients with a very good performance status [12] or with minor restrictions (ECOG 0–1) at the time of participation in the validation study; and PPS group including palliative patients with restricted performance or limited self-care (ECOG 2–3) at the time of participation. ECOG status was determined by the physician responsible for the study. None of the recruited patients had a very poor performance status (ECOG 4). We compared the QLQ-SH-22 self-reported sexual health issues scores between the two groups.

### Statistical analysis

All analyses were performed using SPSS. Descriptive statistics were generated from the sociodemographic and clinical data and presented in frequency tables. Grouping variable was the dichotomized ECOG performance status: fully active patients and those with minor restrictions (ECOG 0–1, GPS) vs. restricted patients (ECOG 2–3, PPS). There was no further adjustment for comparison between ECOG 0–1 and ECOG 2–3. Group differences were evaluated using an independent samples t-test. In case of heterogenous variances the t-test for heterogenous variances was performed. The level of significance was set to 5%. Group differences and effect sizes were calculated according to Cohens d.

## Results

### Sample

A total of 101 patients undergoing oncological treatment with palliative intent with a range of cancers were enrolled. ECOG performance status ranged from 0–3 (Table 1).

**Table 1:** Performance status and groups

ECOG	N, total sample	Group	N subgroups
0	23	GPS	66
1	43		
2	24	PPS	32
3	8		
Missings	3		
Total	101		98

*ECOG* Eastern cooperative oncology Group, Doctors´ assessment of performance status, *GPS* Patient in good performance status, *PPS* Patients in poor performance status

Sociodemographic and clinical data are shown in Table 2. The sample included 44 women (43.6%) and 57 men (56.4%) with an age range from 20 to 80 years. The majority of patients had a sexual partner (76.8%) and lived with a partner or family (68%). Cancer sites are reported in Table 2.

**Table 2:** Gender, Sociodemographic and clinical characteristics (Numbers in whole sample and in Groups- GPS and PPS)

	**Whole sample**		**ECOG 0/1: GPS**		**ECOG 2/3: PPS**	
	**N**	**Proportion**	**N**	**Proportion**	**N**	**Proportion**
**Sex**						
**Female**	44	43.6%	30	45.5%	12	37.5%
**Male**	57	56.4%	36	54.5%	20	62.5%
**Age groups in years**						
20–35 years	3	3%	3	4.5%	0	0.0%
36–50 years	16	15.8%	9	13.6%	6	18.8%
51–65 years	54	53.5%	39	59.1%	15	46.9%
66–91 years	28	27.7%	15	22.7%	11	34.4%
**Living situation**						
Living with partner or family	68	68%	46	70.8%	20	62.5%
Living alone	19	19%	8	12.3%	10	31.3%
Living with others	13	13%	11	16.9%	2	6.3%
Missing	1					
**Sexual partner**						
Yes	76	76.8%	51	78.5%	24	75.0%
No	23	23.2%	14	21.5%	8	25.0%
Missing	2					
**Tumour site**						
Breast	15	14.9%	8	12.1%	5	15.6%
Gynaecologic	13	12.9%	9	13.6%	4	12.5%
Prostate	11	10.9%	5	7.6%	6	18.8%
Other genitourinary	4	4.0%	1	1.5%	3	9.4%
Head and neck	10	9.9%	7	10.6%	3	9.4%
Colorectal	11	10.9%	6	9.1%	5	15.6%
Lung	18	17.8%	13	19.7%	5	15.6%
Brain	2	2.0%	2	3.0%	0	0.0%
Oesophageal, stomach	5	5.0%	4	6.1%	1	3.1%
Hematologic	5	5.0%	5	7.6%	0	0.0%
Other (liver, thyroid, gall bladder)	7	6.9%	6	9.1%	0	0.0%

*ECOG* Eastern cooperative oncology Group, Doctors´ assessment of performance status, *GPS* Patients in good performance status, *PPS* Patients in poor performance status

### Differences in sexual health scores

Group differences concerning the QLQ-SH22 scores and the ECOG performance status are presented in Table 3. There are significant differences between patients with GPS (ECOG 0–1) and patients with PPS (ECOG 2–3) as to their self-reported sexual satisfaction, importance of sexual activity, decreased libido, fatigue and treatment effect on sexual activity. We found moderate to high effect sizes.

**Table 3:** Mean differences, standard deviation between patients with GPS and patients with PPS

**Groups**	**GPS ECOG 0–1***(n* = *66)*		**PPS ECOG 2–3*** (n* = *32)*		***p***	***Cohens´ d***
SHQ22 scores	***M***	***SD***	***M***	***SD***		
Sexual satisfaction	40.94	28.59	28.15	18.54	**0.015**	**0.53**
Sexual pain	13.79	22.22	18.59	24.79	0.386	
Importance of sexual activity	35.59	32.67	50.00	31.26	**0.049**	**0.45**
Troubled by reduced libido	44.25	34.98	23.66	32.42	**0.008**	**0.61**
Worries concerning incontinence	14.88	27.65	29.03	36.25	0.065	
Fatigue	47.53	34.63	66.67	37.71	**0.027**	**0.53**
Treatment effect on sexual activity	44.03	37.99	19.35	36.29	**0.005**	**0.69**
Communication with professionals	6.55	19.51	13.98	24.00	0.146	
Insecurity with partner	66.67	36.51	73.33	33.33	0.444	
Confidence in erection	40.00	37.55	24.07	35.80	0.155	
Body image (male)	62.37	40.13	43.14	42.11	0.125	
Vaginal dryness	20.63	28.82	41.67	42.72	0.137	
Body image (female)	73.91	37.55	63.64	34.82	0.451	

*GPS* Good performance status, *PPS* Poor performance status

Patients with GPS reported significantly more sexual satisfaction than patients with PPS (*p* = 0,015). Further, patients with GPS reported significantly more treatment effects on their sexual activity (*p* = 0.005) and suffered more from decreased libido (*p* = 0.008).

Patients with PPS reported significantly more fatigue (*p* = 0.027), but also attributed significantly more importance to sexual activity than GPS palliative patients (*p* = 0.049).

Patients with PPS and GPS report low levels of communication with health care professionals about sexual health issues; there is no statistical difference in levels of communication for patients with GPS and for patients with PPS (GPS M = 6.55, SD = 19.51 vs PPS M = 13.98, SD = 24.0, *p* = 0.146) s. Table 3.

## Discussion

This observational cohort study explored the sexual health of cancer patients in oncological treatment with palliative intent in relation to their physical performance status and compared patients with GPS versus PPS.

In our analysis we found significant differences between palliative patients with GPS and PPS in terms of their experience of sexual health. These differences concern their self reported sexual satisfaction, importance of sexual activity, decreased libido, fatigue and treatment effect on sexual activity.

Palliative patients with GPS report a significantly higher level of sexual satisfaction. The multi-item *Sexual satisfaction scale* of the EORTC-QLQ-SH22 includes various aspects: satisfaction with the level of sexual desire, the ability to reach an orgasm, communication about sexual issues with a partner, level of intimacy, but also sexual activity and sexual enjoyment. All these aspects together reflect a fulfilling sex life. It is remarkable, that a group of patients in a palliative treatment setting reported a similar level of sexual satisfaction as compared to patients undergoing treatment with curative intent [6]. Patients with GPS report significantly more treatment effects on their sexual activity than patients with PPS and correspondingly, decreased libido is reported as a greater issue by them. These results reflect that sexual activity can be seen as a part of physical performance and is therefore expected to correlate with ECOG status.

In this subgroup analysis, the palliative patients with GPS report significantly less fatigue. Fatigue is a very common symptom among oncological patients: 32 to 90% of all patients with advanced cancer experience fatigue [13, 14]. Our results match these assumptions and confirm clinical experience: poor performance status was associated with more fatigue.

One of the results of our investigation stands out: PPS palliative patients place more importance on sexual activity than GPS palliative patients. Though the PPS group of patients suffers significantly from fatigue and loss of libido, sexual activity retains its importance in their lives. These results concur with the results of Wang et al.´s narrative review of the sexual health needs of cancer patients receiving palliative care [4]. The authors conclude that “sexuality and intimacy remain important parts of many people’s lives regardless of their health”, which underlines the importance of addressing sexual health needs in palliative care. Still, sexuality plays a minor role in the care of terminally ill patients, even though a core principle of palliative care is a holistic approach [15]. It is noteworthy that sexual satisfaction decreases with declining ECOG, although physical functioning (such as confidence to get an erection or vaginal dryness) remain stable. One could interpret these results in relation to multiple losses experienced along the treatment trajectory; that in the face of the loss of various physical functions sexuality may accrue special meaning for patients. Or, as Schopenhauer wrote: “It is usually only loss that teaches us about the value of things” (quote from: “Parerga und Paralipomena, Aphorismen zur Lebensweisheit”).

Our decision to divide and compare our sample according to performance status (ECOG 0 and 1 vs ECOG 2 and 3) was primarily informed by the literature which reports a broad consensus, that ECOG performance status has a decisive influence on prognosis and HRQOL, especially in the palliative context [16, 17]. The evidence for the early introduction of specialist palliative care along the cancer trajectory leads to palliative treatment for patients who are still in good status [18, 19, 20], which could be the reason why a large proportion of the patients with palliative oncological therapy considered in this study showed good general congestion. Perhaps most importantly, groundbreaking advances in oncological therapy in the last decade underpinned our decision, for example, implementation of immunotherapy with immune checkpoint-inhibitors, and image-guided therapy. These therapeutic advances in oncology and radiotherapy provide opportunities for second-, third- and fourth-line- treatments for patients in a palliative situation without severe side-effects [21]. Therefore, there are more and more patients with a good performance status undergoing different types of palliative anticancer treatments and specialist palliative symptom management treatments [22]. As QoL is the main therapeutic goal in palliative treatment, it is of central importance to identify factors that may positively impact an individual’s life.

Both patient groups in our study report a very low level of communication with health care professionals about sexual health issues, and patients with GPS reported even lower levels of communication than patients with PPS. This is in line with earlier findings that there is limited communication with cancer patients about sexuality: patients in palliative care settings wish to have discussions concerning their sexual health with health care professionals, but it is well documented that health care professionals are ill equipped to have such discussions [23, 24, 4]. Williams and Addis report major educational needs among health care professionals concerning communication about patients’ sexual problems. If addressed at all in clinical care, it is usually the physical aspects of sexuality that are spoken of, including physical concerns such as erectile function, vaginal dryness or painful intercourse or menopausal changes [25, 26]. Our results support the need for a much broader and more nuanced discussion about sexuality in the curative as well as in the palliative oncological setting. Previously Vitrano et al. showed that even patients with shorter life expectancy considered it important to talk about and face the issue of sexuality with an experienced professional [27]. Patients in their study had not had that opportunity. Patients in our sample also reported low levels of communication about sexual matters. These results underline, that deficiencies in information provision and adequate discussion of sexual health issues in palliative settings are common. If so, then this is unacceptable and against the holistic principle of palliative care. There is an urgent need for palliative patients’ sexual health needs to be identified and addressed. Clinical use of the EORTC QLQ-SH22 could be a first step to identify the wishes and needs of patients and their partners in relation to sexual issues, and facilitate these important conversations with health care professionals.

### Limitations

This study is not a primary analysis, but a secondary analysis of a large oncology patient cohort from the validation study of the EORTC-QLQ-SH22 with different cancer sites. The limited number of patients with large confidence intervals is a drawback of our study. It is possible that covariates (presence or absence of partners or age) could partially explain our findings, in particular because, in contrast to the procedure in the validation study, we did not exclude patients without a sexual partner or patients who were not sexually active from the analysis. In this secondary analyses is was not possible to assess further information on medication and types of comorbidity, which could as well have a great impact on sexual health, as well. Moreover, we are clearly aware of the fact, that the sample may not be representative of the wide diversity of patients in palliative oncological treatment, as recruitment took place in the context of psychometric assessment in the oncological setting. Thus, non-oncology palliative patients and palliative patients in home care may not be represented in our results.

## Conclusions

Our data strongly supports that sexual health is important to patients receiving oncological treatment with palliative intent, irrespective of their performance status. Patients with GPS and PPS reported sexual problems and PPS patients’ particular high levels of difficulty. Opportunities to discuss sexual issues must be provided to patients. Our findings necessitate the use of appropriate patient reported outcome measures such as the EORTC QLQ-SH22 as a basis for health care professional conversations to address sexual health matters for patients who are receiving palliative intent treatment.

## Data Availability

The datasets used and/or analysed during the current study are available from the corresponding author on reasonable request.

## Abbreviations

ECOG: Eastern Cooperative Oncology Group
EORTC: European Organization for the Research and Treatment of Cancer
GPS: Good performance status
HRQOL: Health-Related Quality of life
PPS: Poor performance status
QLQ-SH22: Quality of Life Questionnaire- Sexual Health
QLG: Quality of Life Group
